# Antibody drug conjugates: hitting the mark in pancreatic cancer?

**DOI:** 10.1186/s13046-023-02868-x

**Published:** 2023-10-25

**Authors:** Nicole L. Wittwer, Michael P. Brown, Vasilios Liapis, Alexander H. Staudacher

**Affiliations:** 1 Translational Oncology Laboratory, Centre for Cancer Biology, SA Pathology, University of South Australia, Adelaide, SA 5000 Australia; 2 Adelaide Medical School, University of Adelaide, Adelaide, SA 5000 Australia; 3 Cancer Clinical Trials Unit, Royal Adelaide Hospital, Adelaide, SA 5000 Australia

**Keywords:** Pancreatic cancer, Antibody drug conjugate, Bystander killing

## Abstract

Pancreatic cancer is one of the most common causes of cancer-related death, and the 5-year survival rate has only improved marginally over the last decade. Late detection of the disease means that in most cases the disease has advanced locally and/or metastasized, and curative surgery is not possible. Chemotherapy is still the first-line treatment however, this has only had a modest impact in improving survival, with associated toxicities. Therefore, there is an urgent need for targeted approaches to better treat pancreatic cancer, while minimizing treatment-induced side-effects. Antibody drug conjugates (ADCs) are one treatment option that could fill this gap. Here, a monoclonal antibody is used to deliver extremely potent drugs directly to the tumor site to improve on-target killing while reducing off-target toxicity. In this paper, we review the current literature for ADC targets that have been examined in vivo for treating pancreatic cancer, summarize current and on-going clinical trials using ADCs to treat pancreatic cancer and discuss potential strategies to improve their therapeutic window.

## Background

Pancreatic cancer is an extremely aggressive cancer with around 500,000 new cases and over 450,000 deaths recorded worldwide in 2020 [1]. This treatment-resistant disease has a rising incidence rate and a stubbornly low and largely unchanged 5-year relative survival rate of approximately 12% [2]. With no reliable biomarkers or methods for early detection and limited effective therapies, pancreatic cancer is predicted to become the second leading cause of cancer related death by 2030 [3].

Within pancreatic cancer, pancreatic ductal adenocarcinoma (PDAC) is the most aggressive and prevalent type, accounting for approximately 90% of all pancreatic cancers. Surgery remains the only curative option for a proportion of PDAC patients, with a 5-year survival rate of 42% in patients with localized disease [2]. Notorious for its late clinical presentation, less than 20% of patients have a tumor confined to the pancreas, with most patients having metastatic disease and potentially curative surgical resection no longer possible. Consequently, chemotherapy remains the best available treatment for metastatic, unresectable PDAC because patients generally present with late-stage disease, have post-surgical recurrence, or both. First line-treatment regimens include the combination of fluorouracil, leucovorin, irinotecan, and oxaliplatin (FOLFIRINOX) [4] and gemcitabine with nab-paclitaxel [5], depending on the patients ECOG performance status and co-morbidities [6–9]. Despite combination chemotherapy advances, the median survival time for pancreatic cancer patients remains at 10–12 months [10]. Given the limited efficacy of current combination chemotherapy regimens, attention has shifted towards targeted therapies and antibody-based approaches to address this highly unmet clinical need.

**Fig. 1:**
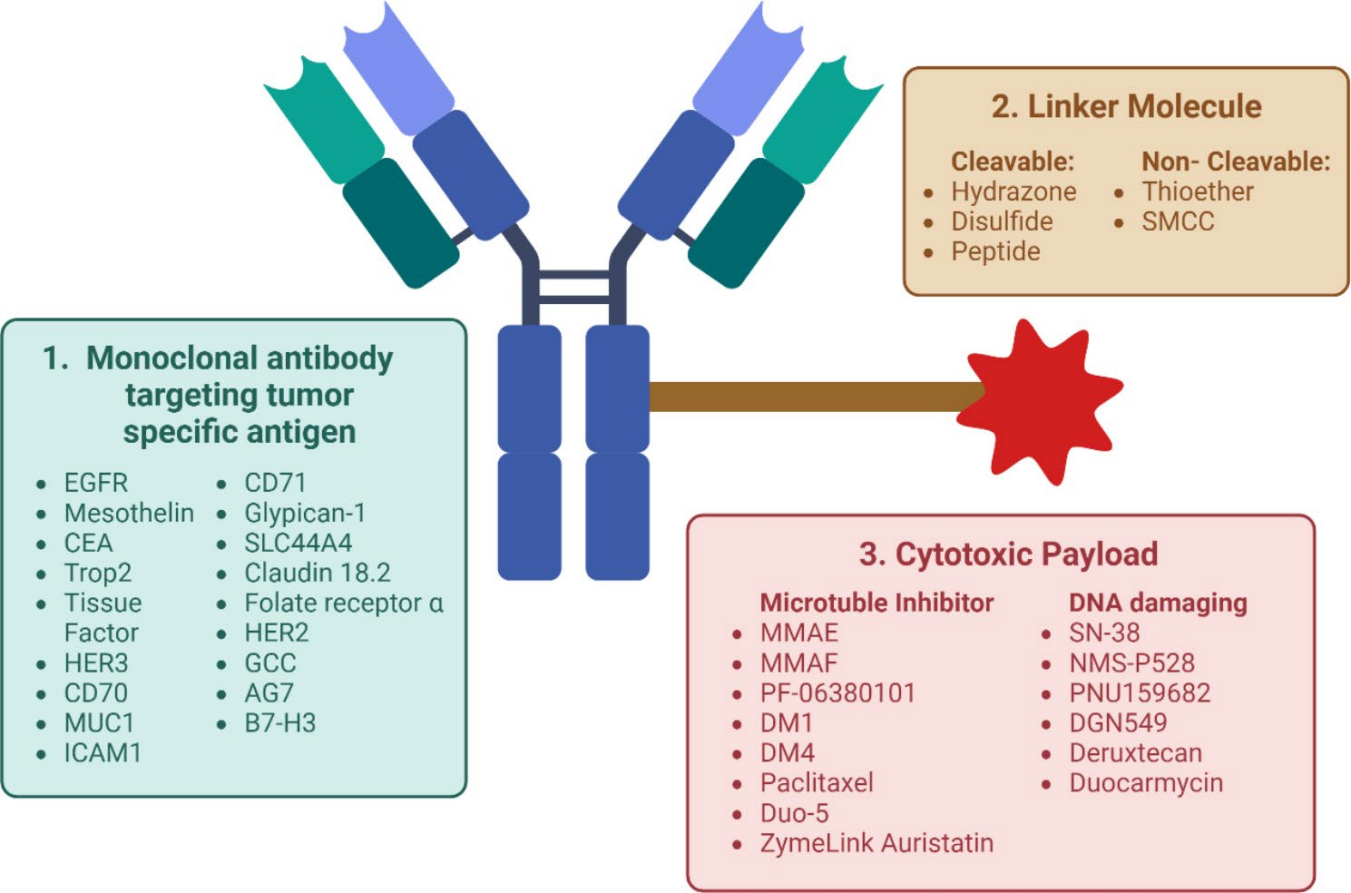
Structure of ADCs and specific components of ADCs in development for targeting pancreatic cancer An ADC is composed of three main components: (1) A monoclonal antibody targeting a tumor specific antigen; (2) a linker molecule and (3) a cytotoxic payload that will kill the target cancer cell. Specific antigens, linkers and payloads being developed for the treatment of pancreatic cancer are listed ***ADC***: *antibody drug conjugate;****B7-H3***: *B7-homolog 3;****CEA***: *carcinoembryonic antigen;****EGFR***: *epidermal growth factor receptor;****GCC***: *Guanylyl Cyclase C;****HER2***: *human epidermal growth factor receptor 2;****HER3***: *human epidermal growth factor receptor 3;****ICAM-1***: *intercellular adhesion molecule 1;****MMAE***: *monomethyl auristatin E;****MMAF***: *monomethyl auristatin F;****MUC1***: *mucin-1;****SMCC***: *succinimidyl-4-(N-maleimidomethyl) cyclohexane-1-carboxylate;****Trop2***: *trophoblast antigen 2.*

Antibody drug conjugates (ADCs) are a unique therapy that combines the direct tumor targeting capability of monoclonal antibodies together with the potency of cytotoxic drugs [11]. Briefly, ADCs are composed of three distinct parts: a tumor-targeting monoclonal antibody (mAb) that specifically binds to a tumor-associated antigen, a high-potency cytotoxic drug, known as a payload, and a chemical linker (Fig. 1). Together, these components direct the tumor-specific delivery of the highly potent cytotoxin, that alone is too toxic to be administered systemically. The targeted intratumoral release of the cytotoxin reduces off-tumor toxicity associated with conventional cytotoxic chemotherapy thus permitting an increased therapeutic window [12, 13]. Each component of the ADC can be customized to satisfy a range of pharmacologic and clinical requirements and, with these improvements in ADC technologies, the ADC field is rapidly expanding with 14 ADCs now approved by the US FDA [11].

Hence, ADCs are designed to ameliorate the high off-tumor toxicity associated with systemic cytotoxic chemotherapy while increasing tumor cell killing. These therapeutic effects have most recently been achieved, largely due to new ADC linker chemistries that stabilize the ADC in the bloodstream and maximize the tumor-specific delivery of the cytotoxic payload [11]. Linkers can be broadly classified as cleavable or non-cleavable, each with their own distinct mechanism of action [14]. Cleavable linkers, such as hydrazone, disulphide and peptide linkers, allow selective cleavage of the ADC within the tumor cell by exploiting environmental differences between healthy and malignant tissues, such as pH or the presence of specific enzymes [15]. Once the ADC is internalized by binding to its tumor-associated antigen, the linker can be cleaved to release the cytotoxic drug directly within the cancer cell. Depending on the payload drug chemistry, the drug can be cell-impermeant resulting in killing of only the targeted cell, or cell-permeant which enables killing of the surrounding, antigen-negative cells in a process called bystander killing. Similarly, a cleavable linker can be cleaved within the tumor microenvironment before cell internalization of the ADC, releasing the drug and also producing bystander killing [16]. Although bystander killing is undesirable for healthy cells, the process could allow eradication of antigen-negative tumor cells that would otherwise not have been targeted by the ADC [17, 18] and could also facilitate the maturation and activation of intratumoral immune cells such as dendritic cells (DCs), which are known to be activated by certain ADC payloads such as maytansines, aurostatins, and pyrrolobenzodiazepine (PBD) dimers [19–21]. Non-cleavable linkers such as thioether and maleimidocaproyl (MMC) linkers, are not susceptible to environmental or enzymatic cleavage and instead are only broken down to release the cytotoxic payload after tumor cell internalization and subsequent antibody degradation in the endosomes and lysosomes [14]. The advantage of non-cleavable linkers includes improved stability and plasma half-life as well as reduced off-target toxicities [15].

The specific targeting of ADCs has allowed for the use of extremely potent drug payloads which are magnitudes of order more potent than standard cytotoxic chemotherapy drugs [15]. Additionally, as only a limited number of drugs can be conjugated to an antibody molecule, and only a fraction of the administered ADC will reach the tumor, extremely potent cytotoxic drugs are required for therapeutic efficacy [22]. Most payloads used in ADCs fall into the classes of tubulin inhibitors or DNA-interacting agents. Tubulin inhibitor payloads, which represent the majority of approved payloads, disrupt the progression of cell division by stabilizing microtubules (auristatin) [23] or de-stabilizing microtubules (maytansinoids) [24]. DNA-interacting payloads can induce cell death by a number of different mechanisms including the induction of DNA double-stranded breaks (calicheamicins) [25], DNA alkylation (duocarmycins) [26], or different modes of DNA cross-linking (PBD dimers) [27] and interference with DNA replication (camptothecins) [28, 29]. DNA-targeting payloads have the advantage of targeting cells at any stage of the cell cycle, and some, such as PBD dimers, are not substrates for multidrug resistance pumps [30].

Currently, FDA-approved ADCs are used in the treatment of hematologic malignancies and solid tumors including breast cancer, urothelial cancer and HER2 + gastric adenocarcinoma [13]. Although there is no FDA-approved ADC for the treatment of pancreatic cancer, interest has grown in their potential application for this disease given the urgent need for new targeted therapies. In this review, we summarize the preclinical studies in which ADCs are being used to target different antigens in pancreatic cancer, followed by a concise overview of clinical trials that are assessing ADCs as a therapy for advanced pancreatic cancer. Lastly, we outline the challenges facing ADC therapy of pancreatic cancer and propose strategies to overcome these challenges and to guide future research directions in this field.

### ADC targets in preclinical models of pancreatic cancer

One of the defining factors in the success of an ADC is the selection of an appropriate tumor-specific antigen. There have been a number of different pancreatic cancer associated antigens that have been investigated in the pre-clinical development of ADCs as well as a range of different ADC payloads and linkers. Common target antigens in pancreatic cancer and ADCs tested in pre-clinical pancreatic cancer models are summarized in Table 1. In this section, we briefly review these pancreatic cancer specific tumor antigens and associated ADCs that have undergone pre-clinical development.

#### Epidermal growth factor receptor

Under normal conditions, epidermal growth factor receptor (EGFR) is expressed largely by epidermal and epithelial cells, and in the islets of Langerhans in normal pancreas. EGFR has been shown to be overexpressed in 30–90% of pancreatic cancer and drives pro-survival and anti-apoptotic pathways [31], thus making it a suitable target for cancer therapy. Moreover, many patients develop resistance to anti-EGFR therapy, indicating that novel EGFR-directed ADCs may help to improve treatment outcomes.

Wong et al. generated low-affinity humanized EGFR antibodies with the assumption that these antibodies would only transiently bind (monovalently) to low EGFR-expressing healthy cells while the antibodies would stably (bivalently) bind to high EGFR-expressing cancer cells. They showed in vitro that the uptake of the low- and high-affinity antibodies by cancer cells was equivalent, however the uptake of low affinity mAbs by normal epidermal cells was reduced. Accordingly, a low affinity EGFR ADC (RN765C), showed potent killing of moderate to high EGFR-expressing cancer cell lines yet showed limited toxicity to human epidermal keratinocytes, and was effective as a single-dose treatment in a pancreatic xenograft model [32].

**Table 1:** Pancreatic cancer associated antigens in ADC pre-clinical development

Target Antigen	ADC name	Linker	Payload	Pre-clinical model	Reference
EGFR	RN765C	Cleavable VC	PF-06380101 (microtubule inhibitor)	BxPC3 pancreatic xenograft	[32]
	RC68	Cleavable VC	MMAE (auristatin)	BxPC3 pancreatic xenograft	[33]
	CTX-MMAE	Cleavable dibromopyridazinedione VC	MMAE (auristatin)	MIA PaCa-2 and PANC-1 pancreatic xenograft	[34]
	LR004	Non-cleavable SMCC	DM1 (maytansinoid)	Capan-2 pancreatic xenograft	[35]
Mesothelin	BAY 94-9343 (Anetumab Ravtansine)	Cleavable (disulfide)	DM4 (maytansinoid)	MIA PaCa-2 (overexpressing mesothelin) pancreatic xenograft Patient-derived pancreatic model (PAXF736)	[18]
	AMA-MMAE	Cleavable VC	MMAE (auristatin)	Capan-2, HPAC, AsPC-1 and HPAF-II pancreatic xenograft	[36]
CEA	Labetuzumab-SN-38	Cleavable	SN-38 (topoisomerase I inhibitor)	Capan-1 pancreatic xenograft	[37]
	α-CEA-680-PTX	Cleavable (disulfide)	Paclitaxel	BxPC3 pancreatic xenograft	[38]
	SAR408701	Cleavable	DM4 (maytansinoid)	N/A	[39]
Trop2	hRS7	Cleavable	SN-38 (topoisomerase I inhibitor)	Capan-1 and BxPC3 pancreatic xenograft	[40, 41]
	RN927C	Cleavable VC	PF-06380101 (microtubule inhibitor)	BxPC3 pancreatic xenograft Patient-derived pancreatic model (Pan0123, Pan0135, and Pan0146)	[42]
Tissue factor	Anti-human ADC and anti-mouse ADC	Cleavable VC	MMAE (auristatin)	Capan-1 and BxPC3 pancreatic xenograft	[43]
	Anti-TF ADC	Cleavable VC	MMAE (auristatin)	TF-expressing murine pancreatic model (mPan)	[44]
	Anti-TF ADC (humanized)	Cleavable VC	MMAE (auristatin)	BxPC3 and HPAF-II pancreatic xenograft	[45]
	SC1-DM1 SC1-MMAE	Non-cleavable Cleavable VC	DM1 (maytansinoid) MMAE (auristatin)	BxPC3 pancreatic xenograft	[46]
	TF-011-MMAE TF-011-MMAF TF-098-MMAE TF-098-MMAF TF-111-MMAE TF-111-MMAF	Cleavable VC Non-cleavable Cleavable VC Non-cleavable Cleavable VC Non-cleavable	MMAE (auristatin) MMAF (auristatin) MMAE (auristatin) MMAF (auristatin) MMAE (auristatin) MMAF (auristatin)	HPAF-II, AsPC-1 and BxPC3 pancreatic xenograft	[47]
HER3	HER3-ADC	Cleavable VC	MMAE (auristatin)	BxPC3 and HPAC pancreatic xenograft and patient-derived pancreatic model (PDX P4604)	[48]
	EV20/NMS-P945	Cleavable	NMS-P528 (DNA alkylating agent)	BxPC3 pancreatic xenograft	[49]
CD70	SGN-75	Non-cleavable	MMAF (auristatin)	CD70 transfected MIA PaCa-2 pancreatic xenograft	[50]
MUC1	SAR566658	Cleavable	DM4 (maytansinoid)	Capan-2 pancreatic xenograft	[51]
	HzMUC1-MMAE	Cleavable VC	MMAE (auristatin)	Capan-2 and CFPAC-1 pancreatic xenograft	[52]
ICAM1	ICAM1-DM1	Non-cleavable SMCC	DM1 (maytansinoid)	PANC-1 pancreatic xenograft	[53]
CD71	CX-2029	Cleavable VC	MMAE (auristatin)	Patient-derived pancreatic model (PA6237)	[54]
Glypican-1	GPC1-ADC	Cleavable VC	MMAE (auristatin) MMAF (auristatin)	BxPC3 and BxPC3 GPC-1 Knockout pancreatic xenograft Patient-derived pancreatic model (PK645 PDAC, PK565 PDAC, PK175, and KPK1)	[55–57]
SLC44A4	ASG-5ME	Cleavable VC	MMAE (auristatin)	Patient-derived pancreatic model (AG-Panc3 (orthotopic), AG-Panc4 and AG-Panc2)	[58]
DR5	Oba01	Cleavable VC	MMAE (auristatin)	MIA PaCa-2 and PL45 pancreatic xenograft Patient-derived pancreatic model (PA1266 and PA1198)	[59]

**ADC**: antibody drug conjugate; **CEA**: carcinoembryonic antigen; **DR5**: death receptor 5; **EGFR**: epidermal growth factor receptor; **GPC-1**: glypican-1; **HER3**: human epidermal growth factor receptor 3; **ICAM-1**: intracellular adhesion molecule 1; **MMAE**: monomethyl auristatin E; **MMAF**: monomethyl auristatin F; **MUC1**: mucin-1; **SMCC**: succinimidyl-4-(N-maleimidomethyl) cyclohexane-1-carboxylate; **Trop2**: trophoblast antigen 2; **VC**: valine-citrulline

A different, humanized anti-EGFR mAb (RC68) was conjugated to MMAE using two different types of cleavable VC linker: a maleimide-type linker (MC) which binds the drug to single thiol groups on the mAb, or a novel PY linker capable of covalently linking the drug with two thiol groups to bridge two heavy or a heavy and a light chain within the mAb. While the PY linker provided increased serum stability in vitro, both ADCs showed similar binding affinity and dose-dependent anti-tumor activity in a pancreatic cancer xenograft model, which was superior to gemcitabine treatment [33].

An EGFR ADC was generated using a dibromopyridazinedione linker, which bridges the inter-sulfide bonds of the mAb, to generate a more uniform drug to antibody ratio (DAR) of approximately 4 and improve serum stability. This ADC contained the MMAE payload and was able to overcome EGFR mAb-resistance associated with KRAS mutation, which is present in almost all pancreatic cancers [34].

The humanized EGFR-targeting antibody LR004 showed anti-tumor activity when given as a single treatment in MIA PaCa-2 and PANC-1 pancreatic tumor models, with the response potentiated when multiple doses were given [35].

#### Mesothelin

The low expression of mesothelin on healthy tissues and its overexpression in malignancy, particularly carcinoma, has made it an promising target for antibody-based cancer treatments [60]. Mesothelin is expressed in > 90% of pancreatic cancers [61, 62], making it an attractive target for therapy.

An anti-mesothelin targeting ADC, BAY 94-9343, was used for the treatment of pre-clinical cancer models including pancreatic cancer [18]. In this study, mesothelin was overexpressed on the human pancreatic cancer cell line MIA PaCa-2, and the resulting cell line was sensitized to the anti-mesothelin ADC. In a subcutaneous pancreatic cancer model, a dose-dependent response to the ADC was observed, with a complete response seen at the highest administered dose delivered across three injections. In a PDX model of pancreatic cancer (PAXF736), this ADC given at the maximum dose resulted in transient regression of tumors and was superior to the standard treatment of gemcitabine [18].

AMA-MMAE has been used in treating a range of cancers including pancreatic cancer [36]. Interestingly, although the four pancreatic cancer cell lines examined showed appreciable mesothelin expression in vitro, the response to the ADC varied in vivo and was independent of the expression levels of mesothelin. This was not due to downregulation of mesothelin because its expression was confirmed ex vivo; however, PET imaging using ^89^Zr-anti-meosthelin ADC showed reduced tumor uptake in the unresponsive pancreatic cancer models. This result was unlikely to be due to shedding of mesothelin from the cancer cells, but rather lower antigen internalization for the less-responsive pancreatic cancer models, thereby reducing the amount of active drug released [36].

#### Carcinoembryonic antigen (CEA)

The carcinoembryonic antigen (CEA), also known as carcinoembryonic antigen-related cell adhesion molecule 5 (CEACAM5) and CD66e, is a cell-surface adhesion protein and its expression is limited to the gastrointestinal tissue during fetal development. However, CEA is highly expressed in a variety of different cancer types, including pancreatic cancer [63].

A humanized CECAM5 mAb (labetuzumab) was conjugated with SN-38, a camptothecin which inhibits topoisomerase I, resulting in cell cycle arrest and apoptosis. As a free drug, SN-38 is detoxified back to its glucuronide form, then reconverted back in the intestine, causing delayed toxicity [64]. Therefore, improved methods of drug delivery specific to the cancer were required. Govindan et al. prepared labetuzumab with SN-38 and a cleavable linker and tested the ADC in a variety of in vivo models including pancreatic cancer [37]. They showed that repeated treatment with labetuzumab-SN-38 was well tolerated and significantly increased survival in a human pancreatic cancer xenograft model which was superior to mice treated with equimolar amounts of free antibody and free drug.

Knutson et al. used a CEA-targeting mouse mAb conjugated with a near-infrared fluorophore and paclitaxel and showed that this ADC could be tracked in vitro and in vivo and was internalized by BxPC-3 pancreatic cancer cells [38]. When used at equivalent molar concentration in vitro, the ADC was less effective compared to the free paclitaxel; however, when used in vivo, the ADC targeted the tumor and reduced tumor growth compared to an equimolar amount of free drug when given as 5 injections, 3 days apart, although statistical significance was not reached.

A humanized CEACAM5 mAb conjugated via a SPDB linker to DM4 (SAR408701) showed efficacy in vitro against pancreatic cancer cell lines [39]. Although not examined in vivo using pancreatic cancer models, the ADC was shown to be effective as single and multiple treatments for PDXs of colon, lung and stomach cancer [39].

#### Trop2

Trophoblast antigen 2 (Trop2) is a transmembrane glycoprotein with varied expression in healthy tissues and is overexpressed in some cancer types where its signaling results in increased proliferation, self-renewal and invasion [65, 66]. Trop2 is overexpressed in over half of pancreatic cancers and its elevated expression correlates with decreased overall survival, metastasis, and worse progression-free survival [67].

A humanized anti-Trop2 ADC (hRS7) was effective in controlling tumor growth in two human pancreatic cancer xenograft models when given as eight treatments, whereas the treatment with the pro-drug irinotecan (given at a SN-38-dose equivalent) had no significant effect on tumor growth. Interestingly, a control ADC with the same linker and payload also showed significant tumor growth inhibition compared to irinotecan alone, suggesting induction of bystander killing by the control ADC [16].

Sharkey et al. used a novel strategy of combining an ADC with a radiolabelled antibody (radioimmunotherapy, RIT) in a variety of pre-clinical models of human cancer, including pancreatic cancer [41]. Using a similar 8-treatment strategy as above, hRS7-SN-38, an anti-Trop2 ADC showed some anti-tumor activity, but with only a 10% cure rate. Administration of a single dose of 2.8 MBq (75 µCi) or 4.8 MBq (130 µCi) of Yttrium-90-labelled humanized anti-mucin PAM4 antibody resulted in 10% and 50% cure rate, respectively. The combination of both hRS7-SN-38 and ^90^Y-hPAM4 (starting on the same day) resulted in 40% and 90% cure rate at 2.8 MBq and 4.8 MBq ^90^Y-hPAM4, respectively. Furthermore, complete cures could be induced with a combination approach of chemotherapy (gemcitabine), RIT and ADC.

Modification of the humanized anti-Trop2 antibody RN927C, where the C-terminal lysine of the heavy chain was replaced with a specific tag, allowed for exact conjugation of the linker and drug payload, resulting in a DAR of exactly 2. This ADC showed a dose-dependent reduction in tumor growth in a pancreatic xenograft model and three different pancreatic PDXs models, which was superior compared to conventional gemcitabine chemotherapy [42].

#### Tissue factor

Tissue factor (TF), also known as CD142, activates the coagulation cascade in response to tissue damage and trauma in healthy tissues. TF is overexpressed in cancer, where it increases tumor cell growth likely through increased angiogenesis, and increases metastasis leading to poorer prognosis [68].

Anti-mouse and anti-human TF mAbs were used to generate TF-targeting ADCs [43], and the cytotoxicity of the anti-human TF-ADC correlated to expression of TF on pancreatic cell lines in vitro. These ADCs showed a dose-dependent anti-tumor response in vivo in a human pancreatic cancer xenograft model expressing high levels of TF. Interestingly, both the anti-mouse-TF and isotype control ADC showed some anti-tumor response, although weaker than that seen with the anti-human ADC. Tumor analysis showed high uptake of the anti-human ADC within the periphery of the tumor, while the anti-mouse ADC and control ADC showed reduced tumor uptake and were in the tumor stroma. In a low TF-expressing xenograft model, both the anti-human and anti-mouse TF ADC showed equivalent anti-cancer activity, suggesting that perfusion of the ADCs into the tumor is sufficient to elicit some tumor response. This is likely due to cleavage of the ADC in the tumor microenvironment and the permeable nature of the drug inducing bystander killing.

Tsumura et al. generated anti-mouse TF ADCs with a VC linker and MMAE payload using the standard maleimide-based conjugation or through bis-alkylation conjugation (bisAlk), which can re-bridge the reduced interchain disulfide bonds [44]. Using a TF-expressing murine pancreatic cancer cell line (mPan), both ADCs showed similar binding in vitro and similar potency against mPan TF-expressing cells but not wild-type mPan cells. When examined in vivo, both ADCs were well tolerated and active against mPan TF-expressing subcutaneous xenografts. The bisAlk ADC showed some improvement in tumor control at lower administered dose, and significantly reduced tumor growth in the mPan WT xenograft model, which had lower TF expression. This ADC was also successful in controlling tumor growth in an orthotopic model using mPan TF-expressing cells. This effect was due, in part, to a reduction in TF-expression and killing of endothelial cells.

A humanized, anti-human TF mAb demonstrated specific cytotoxicity to human pancreatic cancer cells in vitro, but not to the same cell line which had TF knocked out. The mAb alone showed no therapeutic effect in subcutaneous pancreatic cancer xenograft models but, when given as an ADC in three injections, was able to attenuate tumor growth. This response was specific for TF-expressing tumor cells as there was no response to the ADC in a TF-KO xenograft model. This ADC was also effective in an orthotopic and peritoneal dissemination model of pancreatic cancer [45].

A mouse mAb to human TF (SC1) reduced pancreatic cancer cell migration in vitro, and reduced tumor growth in vivo. ADCs of this antibody using SMCC-DM1 or VC-MMAE payloads were generated and showed potency against pancreatic cancer cells in vitro. The SC1-MMAE ADC and a humanized variant of the ADC, given once a week for 4 weeks showed a dose-dependent reduction in tumor growth. This treatment was well tolerated, with the equivalent amount of docetaxel required to induce the same tumor growth inhibition, resulting in toxicities that led to substantial weight loss [46].

Breij et al. generated a panel of human TF-specific human antibodies and screened them for their ability to induce antibody dependent cellular cytotoxicity (ADCC) and inhibit TF:FVIIa intracellular signaling (likely preventing production of proangiogenic factors, cytokines and adhesion molecules) while having minimal impact on TF coagulant activity. These antibodies alone showed some benefit when given as a prophylactic treatment shortly after tumor cell injection in pancreatic cancer xenograft models yet had little effect when used to treat established tumors. ADCs were next generated by conjugating the TF antibodies to MMAE or MMAF using a VC linker. Across the three mAb clones studied, the ADC with the MMAE payload showed superior activity compared to MMAF in regressing tumor growth in a pancreatic xenograft model, and controlled tumor growth when given as a single treatment or as multiple treatments. This ADC was used to treat a pancreatic PDX model with heterogeneous expression of TF, and the ADC induced complete regression in these mice [47].

#### HER3

HER3 is part of the human epidermal growth factor (HER) family, which has no or minimal intrinsic activity; however it is overexpressed in a variety of cancers and is associated with increased metastasis, poorer response to therapy and decreased survival [69]. Cancer associated fibroblasts secrete neuregulin-1 (NRG1), which activates the HER3 pathway and promotes pancreatic tumor growth in vivo [70].

Bourillon et al. generated a HER3 targeting ADC using a non-NRG1 competitive mouse mAb (9F7-F11) conjugated to MMAE using a VC cleavable linker. As this ADC not only induced apoptosis but also caused cell cycle arrest in G2/M phase, the authors reasoned that prior treatment with this ADC would radiosensitize cancer cells. Indeed, this approach did sensitize two xenograft and one PDX model of pancreatic cancer to external beam radiation both in vitro and in vivo, due to reduced proliferation, increased DNA damage and apoptosis [48].

A therapeutic humanized anti-HER3 antibody (EV20) conjugated through a cathepsin-cleavable linker to the DNA minor groove-binding agent NMS-P528, was shown to be effective in a variety of xenograft models, including pancreatic cancer [49]. A single, high dose (40 mg/kg) of the ADC was able to control the growth of a well-established pancreatic cancer xenograft and was well tolerated with no significant changes in hematologic profile.

#### CD70

CD70 is a costimulatory molecule and member of the tumor necrosis factor superfamily. It is expressed on activated B- and T-cells, limiting its expression to lymphoid tissues. It is overexpressed in some cancers, where it interacts with its ligand, CD27, to induce proliferation and can dampen anti-tumor immune response [71]. CD70 is expressed in approximately 25% of pancreatic cancers and its elevated expression is correlated with increased metastasis and poorer response to chemotherapy [50, 72].

A humanized CD70 targeting (SGN-75) ADC was effective at killing a CD70-expressing ovarian cancer cell line, but it had no effect on the pancreatic cancer cell lines tested (Panc-1 and MIA PaCa-2) due to low or no CD70 expression on these lines, respectively. A CD70-expressing MIA PaCa-2 line was generated and was shown to be sensitive to SGN-75 in vitro. When given as four injections SGN-75 was able to significantly decrease tumor growth when the same line was used as a xenograft model [50].

#### MUC1

Mucin 1 (MUC1) is normally expressed on all epithelial cells and it, along with other mucin glycoproteins, form the major macromolecular component of mucus. MUC1 expression in normal pancreas is minimal, whereas its expression is increased in approximately 90% of pancreatic cancers [73].

The CA6 sialoglycotope of MUC1, a MUC1 epitope with limited expression on healthy tissue and overexpressed in epithelial cancers, was shown to be expressed by 76% of pancreatic cancers using the humanized antibody, huDS6 [51]. The antibody was conjugated with DM4 using a SPDB cleavable linker and this ADC showed a dose-dependent reduction in tumor growth when given as a single dose in a pancreatic xenograft model.

A humanized antibody (HzMUC1) which binds to an alternate MUC1 epitope, the SEA domain, was conjugated with a cleavable VC linker to MMAE. This ADC was shown to induce G2/M arrest and cause apoptosis in pancreatic cells in vitro. This was mirrored in vivo, with three doses of the ADC significantly decreasing the growth of pancreatic cancer xenografts by reducing proliferation and inducing apoptosis within the tumor [52].

#### ICAM1

Intracellular adhesion molecule 1 (ICAM1, also known as CD54) is a multifunctional cell surface protein of the immunoglobulin superfamily which is overexpressed in cancers. The KRAS G12D mutation, which accounts for approximately 30% of all KRAS mutations in pancreatic cancer, drives expression of ICAM1 by acinar cells in vivo a mouse model, attracting macrophages to accelerate formation of pre-cancer pancreatic lesions [74]. This marker was shown to ubiquitously expressed by 4 pancreatic cancer cell lines examined, with a range of 3 × 10^5^ to 10^6^ molecules per cell, while expression was near absent on normal human pancreatic duct epithelial cells. This was confirmed using healthy pancreatic and pancreatic cancer tissue microarrays, with no ICAM1 detected in normal pancreas and high ICAM1 expression in pancreatic cancer, with ICAM1 expression correlating with stage of disease and survival. The authors demonstrated that while an ICAM1-targeting antibody alone did not alter cell proliferation, it did however inhibit cell migration in vitro. A range of ICAM1 ADCs were generated with different linkers and payloads including an ADC with non-cleavable linker and low cell permeable drug (ICAM1-DM1), which showed the greatest potency against pancreatic cancer cells while not altering the viability of normal human pancreatic duct epithelial cells. When examined in an orthotopic pancreatic cancer model, the ICAM1 ADC significantly reduced tumor growth in part by reducing tumor cell proliferation. This treatment also reduced the level of metastatic disease [53].

#### CD71

CD71, also known as transferrin receptor 1 is overexpressed by most tumors, however its expression in healthy tissues has limited it targeting capabilities. To bypass this Singh et al. developed a novel probody drug conjugate targeted to CD71. The probody composed of the CD71-targeting antibody CX-051, contained a masking peptide which prevented the antibody from binding to its target. The masking peptide was associated with the antibody via a protease-cleavable linker which, when cleaved within the tumor microenvironment, would free the masking peptide, allowing the antibody to bind to CD71. The probody was conjugated to MMAE via a VC linker and showed efficacy in a variety of different models, including pancreatic cancer [54].

#### Glypican-1

Glypican-1 (GPC1) is a heparan sulfate proteoglycan which is bound to the plasma membrane via glycosylphosphatidylinositol. GPC1 expression is absent or low in normal healthy pancreas and is overexpressed in pancreatic cancer, where its elevated expression is correlated with poorer survival [75].

Tsujii and colleagues showed that the majority of pancreatic cancer patients express GPC1 on both the tumor and stromal cells, particularly cancer-associated fibroblasts (CAFs). They generated an ADC targeting human GPC1 conjugated with MMAF or MMAE via a VC linker. Both ADCs were shown to be effective in directly killing GPC1-positive pancreatic cells in vitro and in vivo [55–57]. In a PDX model with stromal-positive and cancer-negative GPC1 expression, the MMAE ADC outperformed the MMAF conjugate, which was attributed to processing of the ADC by CAFs, resulting in release of MMAE which was pumped out of the cells via the drug exporter MDR-1 and killed surrounding bystander cells [56]. Using a mixed model of GPC-1 expressing and KO pancreatic cancer cell line, it was shown that a humanized version of the MMAE ADC could significantly reduce tumor growth, while the ADC had no effect KO pancreatic cancer xenograft alone [57].

GPC-1 has become a controversial biomarker in pancreatic cancer. GPC-1 is present in two forms: membrane-bound protein and as a soluble fragment due to shedding or cleavage. In liquid biopsies, circulating exosomes were found to be highly GPC-1 positive in patients with PDAC [76] and correlated with disease burden [77]. However, it has also been shown that extracellular vesicles from patients with benign pancreatic disease also exhibit high GPC-1 expression, bringing in to question its utility as a diagnostic biomarker [78]. These conflicting results highlight the need for more research into GPC-1 as a prognostic marker in pancreatic cancer. As ADCs undergo intracellular processing it is likely that the ADC will only act on the membrane bound GPC-1 protein, however there is potential that the ADC could bind to the soluble fragment found in circulating exosomes, which may limit the availability of ADC to target the tumor. To address this Tsujii and colleagues analyzed the efficacy of their ADC in the presence of recombinant human GPC-1 and showed minimal inhibitory effects at concentrations equivalent to circulating GPC-1 in PDAC patients [56].

#### SLC44A4

SLC44A4, also known as CTL4, is a choline transport-like protein. This protein is overexpressed in epithelial cancers, including 90% of pancreatic cancers, with limited expression in healthy tissues. An ADC comprising anti-SLC44A4 antibody conjugated to MMAE by a VC linker (ASG-5ME) controlled tumor growth in multiple tumor models, including subcutaneous and orthotopic PDX models of pancreatic cancer [58].

#### Death receptor 5 (DR5)

Tumor necrosis factor-related apoptosis-inducing ligand (TRAIL) receptor, death receptor 5 (DR5) shows little to no expression in normal cells but is over-expressed on a range of cancers including pancreatic cancer [79]. A DR5 targeting ADC (Oba01) has recently shown promising pre-clinical anti-tumor activity both as a monotherapy and in combination with gemcitabine in cell-line derived and patient-derived pancreatic cancer xenograft models [59]. While both treatments showed efficacy as standalone agents, the two drugs were able to synergise to significantly prolong tumor control.

This large body of pre-clinical work in the development of ADCs for the treatment of pancreatic cancer has yielded largely positive results. ADCs have demonstrated efficacy in both xenograft and patient-derived pancreatic tumor models and this has been seen with a range of ADCs encompassing different target antigens, linkers and payload combinations. This pre-clinical evidence supports the clinical investigation of ADCs as a treatment modality for pancreatic cancer.

### ADCs in clinical development for pancreatic cancer

A number of early phase clinical trials have taken place investigating ADCs for the treatment of pancreatic cancer starting from 2010. The data available from www.clinicaltrials.gov at the writing of this paper shows that there are trials investigating several different tumor-associated antigens including mesothelin, Trop-2, TF, human epidermal growth factor receptor 2 (HER2) and Guanylyl Cyclase C (GCC) in pancreatic cancer. Additionally, these trials are investigating different payloads including auristatins, maytansinoids and topoisomerase I inhibitors and include both cleavable and non-cleavable linkers. A list of these trials is presented in Table 2. Despite this, the results from only a minority of these clinical studies have been published to date. The next section summarizes the trials with data available in either published manuscript or abstract form.

Anetumab ravtansine (AR) is an ADC consisting of a fully human IgG1 mAb directed toward the tumor-associated antigen mesothelin and is conjugated to the maytansine derivative DM4 via a cleavable linker. AR was first investigated in clinical trial NCT01439152 a large phase I, open-label, multicenter, dose-escalation and dose-expansion study in mesothelin expressing solid tumors, including nine pancreatic cancer patients [80]. Three pancreatic cancer patients enrolled in the study showed stable disease. This was then followed up with clinical study NCT03023722, a multicenter, non-randomized, phase II study evaluating the efficacy of AR in mesothelin-expressing advanced pancreatic cancer [81]. The results from this study were reported on ClinicalTrials.gov in 2021, showing that of the fourteen pancreatic cancer patients enrolled in the study, two achieved stable disease and the median time to progression was 63.5 days. Pancreatic cancer patients were also included in the multi-indication phase 1b study of AR, clinical trial NCT03102320 [82]. The study was completed in 2022, but the results are not yet published. Further clinical evaluation of AR in pancreatic cancer is currently underway in a phase I study evaluating AR in combination with checkpoint inhibition and gemcitabine in advanced pancreatic cancer (NCT03816358). At data cut off (22/01/2022), thirty three patients were enrolled across three arms, investigating different combinations with AR [83]. Arms 1 and 2, which evaluated AR in combination with nivolumab (anti-PD-1 mAb) and nivolumab + ipilimumab (anti-CTLA4 mAb) respectively, observed stable disease in two participants each (2/9 for Arm 1 and 2/8 for Arm 2). In Arm 3, where patients received AR in combination with nivolumab and gemcitabine, stable disease was observed in all eight patients enrolled in the cohort. Given the disease control rate and tolerability, Arm 3 will be tested in an expanded cohort, with these studies currently ongoing.

Sacituzumab govitecan (SG) targets tumor-associated Trop-2, which is over-expressed on a range of solid tumors including pancreatic cancer, and is currently FDA approved for use in triple negative breast cancer [15]. This ADC, which consists of a humanized anti-Trop2 IgG1 mAb conjugated to SN-38 via a cleavable linker, was investigated in the phase I/II study, NCT01631552. This study involved a large cohort of patients with a range of malignancies including sixteen patients with pancreatic cancer. While efficacy was seen in several cancer cohorts, results in pancreatic cancer were not as promising. Seven of the sixteen pancreatic cancer patients (43.8%) had stable disease post therapy, but the progression-free survival was only 2 months and median overall survival was 4.5 months [84]. While further trials of SG are underway for other cancer types, no further evaluation appears to be planned for pancreatic cancer. Interestingly another Trop-2 targeting ADC, datopotamab deruxtecan (Dato-DXd, DS-1062a), which is also conjugated to a potent DNA topoisomerase I inhibitor is under investigation in a phase I/II clinical trial NCT04644068 in combination with the oral PARP inhibitor AZD5305 for a range of solid cancers including pancreatic cancer. The trial is ongoing, and results have not yet been published.

**Table 2:** ADCs investigated in clinical trials for pancreatic cancer

Trial Number	Phase	ADC name	Target	Linker	Payload	Status
ADC Monotherapy						
NCT05043987	Phase I	CPO102	Claudin 18.2	Cleavable	MMAE (auristatin)	Not yet recruiting
NCT05525286	Phase I/II	SOT102	Claudin 18.2	Non-cleavable	Anthracycline PNU159682	Recruiting
NCT05156866	Phase I	TORL-2-307-ADC	Undisclosed	Undisclosed	Undisclosed	Recruiting
NCT05498597	Phase I	AMT-151	Folate Receptor alpha	Undisclosed	Undisclosed	Recruiting
NCT04659603	Phase II	Tusamitamab Ravtansine	CEACAM5	Cleavable	DM4 (maytansinoid)	Recruiting
NCT03602079	Phase I/II	A166	HER2	Cleavable	Duo-5 (anti-microtubule agent)	Active, not recruiting
NCT01631552	Phase I/II	Sacituzumab Govitecan-hziy	Trop-2	Cleavable	SN-38 (topoisomerase I inhibitor)	Completed
NCT01166490	Phase I	ASG-5ME	SLC44A4	Cleavable	MMAE (auristatin)	Completed
NCT03023722	Phase II	Anetumab Ravtansine	Mesothelin	Cleavable	DM4 (maytansinoid)	Completed
NCT02999672	Phase II	Trastuzumab Emtansine	HER2	Non-cleavable	DM1 (maytansinoid)	Completed
NCT01577758	Phase I	TAK-264 (MLN0264)	GCC	Cleavable	MMAE (auristatin)	Completed
NCT02202785	Phase II	TAK-264 (MLN0264)	GCC	Cleavable	MMAE (auristatin)	Terminated
NCT03449030	Phase I	TAK-164	GCC	Cleavable	DGN549 (DNA alkylating agent)	Terminated
NCT02908451	Phase I	AbGn-107	AG7 antigen	Proprietary linker	DM4 (maytansinoid)	Terminated
ADC in combination with oral PARP inhibitor AZD5305						
NCT04644068	Phase I/II	Trastuzumab Deruxtecan Datopotamab Deruxtecan	HER2 Trop2	Cleavable	Deruxtecan (topoisomerase I inhibitor)	Recruiting
ADC in combination with checkpoint inhibition						
NCT05293496	Phase I	MGC018	B7-H3	Cleavable	Duocarmycin analog	Recruiting
NCT04925284	Phase I	XB002	Tissue factor	Proprietary linker	ZymeLink Auristatin	Recruiting
NCT03816358	Phase I	Anetumab Ravtansine	Mesothelin	Cleavable	DM4 (maytansinoid)	Active, not recruiting

**ADC**: antibody drug conjugate; **B7-H3**: B7-homolog 3; **CEACAM5**: carcinoembryonic antigen-related cell adhesion molecule 5; **GCC**: Guanylyl Cyclase C; **HER2**: human epidermal growth factor receptor 2; **MMAE**: monomethyl auristatin E

The ADC XB002, targets TF which is overexpressed by a number of solid tumors including pancreatic cancer. XB002 is composed of a TF-directed human antibody conjugated to a novel cytotoxic payload, ZymeLink Auristatin via a proprietary linker and is currently being investigated in the open-label, multicenter, first-in-human phase I trial NCT04925284 [85]. Preliminary results of the ongoing trial have shown no objective responses, however at time of reporting (October 2022) three of the nineteen patients with stable disease remain on treatment, one of whom has pancreatic cancer [86]. The trial is ongoing with an expansion cohort planned, testing XB002 as both a monotherapy and in combination with nivolumab.

TAK-264 (formerly MLN0264) consists of a fully human IgG1 monoclonal anti-GCC antibody conjugated via a protease-cleavable linker to MMAE. TAK-264 was initially tested in a phase 1, first-in-human study (NCT01577758) which evaluated its efficacy in advanced, GCC-expressing gastrointestinal malignancies [87]. Two patients with pancreatic cancer were included in the study and both patients had durable stable disease. As a result of these promising early signs of clinical benefit in pancreatic cancer patients, TAK-264 was investigated further in a phase II study, NCT02202785. This study, which enrolled forty three pancreatic cancer patients, achieved an objective response rate of 3%, with one patient achieving a partial response [88], and an additional nine patients having stable disease. Patients in the study received a median of 2 (range 1–10) treatment cycles, with thirty six patients discontinuing the trial due to disease progression. Based on the interim efficacy results, stage 2 of the study was not initiated, and the trial was terminated early due to futility. The authors did not find correlation between tumor antigen expression and ADC efficacy and suggest that failed internalization of the ADC and/or poor penetration of the ADC into the tumor may account for the limited efficacy of this ADC in pancreatic cancer. A subsequent ADC, TAK-164, was developed based on the same GCC antibody using an alternative DNA alkylating payload. This Phase I study NCT03449030, was open to patients with GCC expressing pancreatic cancer, however no pancreatic cancer patients were recruited into the study which was terminated due to insufficient clinical benefit [89].

ASG-5ME is an ADC consisting of a human IgG2 mAb which targets the solute carrier receptor SLC44A4 conjugated to MMAE via a cleavable linker. NCT01166490 was a phase 1, dose-escalation, multicenter study which recruited patients with advanced pancreatic and gastric cancers, thirty five of whom were pancreatic cancer patients [90]. A disease control rate of 33% was observed at maximum tolerated dose with a partial response in one patient with pancreatic cancer. The median duration of disease control was 2 months. Although ASG-5ME was generally well tolerated with a toxicity profile similar to that observed with other MMAE-containing ADCs, there was limited evidence of antitumor activity with an objective response rate similar to conventional systemic chemotherapy. The authors of the study confirmed SLC44A4 antigen expression on patient’s tumors but suggested that further investigation of this ADC is not warranted. It was postulated that characteristics of the antigen itself such as internalization rate, intracellular trafficking, and localization, may contribute to the limited effectiveness of the ADC in patients.

AbGn-107 is an ADC directed against the AG-7 antigen, a Lewis A-like glycol-epitope which is overexpressed by several gastrointestinal tumors including pancreatic cancer. AbGn-107 is composed of a humanized IgG anti-AG-7 antibody conjugated to a dolastatin analogue via a proprietary cleavable linker and has been evaluated in a phase Ia, first-in-human dose escalation study (NCT02908451). Thirty five patients, which included twenty pancreatic cancer patients, were enrolled in the study across six dose levels [91]. The best response was two patients with stable disease across two different dosing regimens, however it is not clear if these were pancreatic cancer patients. Although AbGn-107 was well tolerated and had preliminary evidence of effectiveness, no further data has been published for this ADC in pancreatic cancer.

Trastuzumab emtansine (T-DM1) is an ADC that targets HER2 and is conjugated to the maytansinoid DM1 via a non-cleavable thioether linker. T-DM1 was originally FDA approved in 2013 for the treatment of metastatic HER2-positive breast cancer [15]. T-DM1 was investigated in a multicenter, non-randomized, phase II study, NCT02999672, for the treatment HER2 overexpressing metastatic pancreatic cancer and cholangiocarcinoma. The results from this study were reported on ClinicalTrials.gov in 2019, showing that of the seven participants enrolled in the study, one participant achieved a partial response with progression free survival of 2.58 months. The trial was terminated early, and no further data have been published for this ADC in pancreatic cancer.

Taken together these clinical studies suggest that despite great success of ADCs in the treatment of hematological malignancies and breast cancer, the same potential has not yet been realized in clinical trials for pancreatic cancer. This is likely due to the aggressive and treatment refractory nature of this disease, which presents many challenges. However, as our understanding of the unique pancreatic cancer microenvironment improves, these hurdles can be addressed and overcome to improve the success of ADCs in the clinic.

### Current hurdles and future directions of ADC therapy in pancreatic cancer

**Fig. 2:**
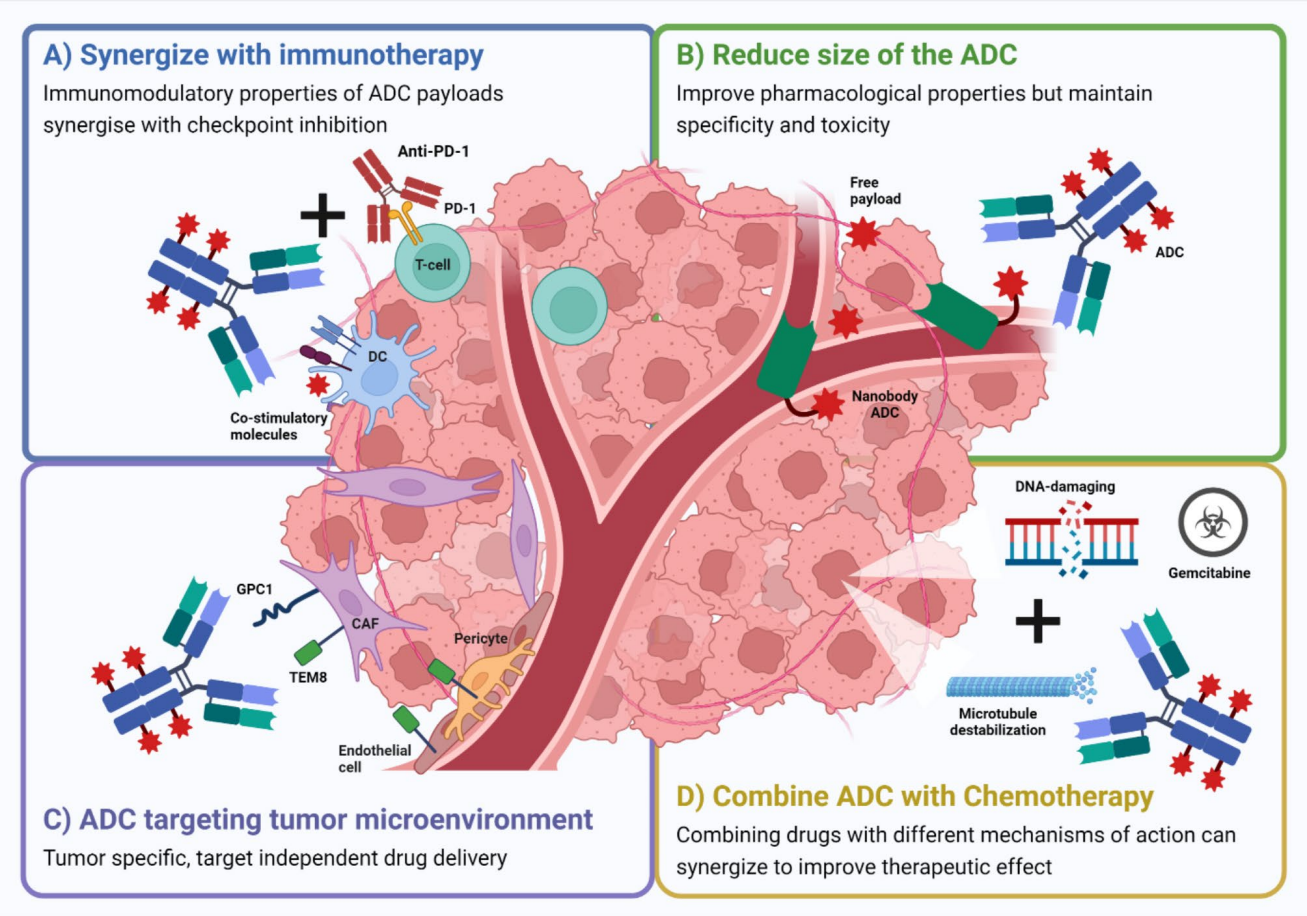
Strategies to improve ADC efficacy in pancreatic cancer Schematic representation of different approaches being undertaken to improve efficacy of ADC therapy in pancreatic cancer. **(A)** Synergistic interaction between the ADC and immunotherapy may enhance therapeutic response. ADC payloads are known to exhibit immunomodulation through upregulation of co-stimulatory molecules on dendritic cells, which can synergize with checkpoint inhibition to enhance effector T-cell function. **(B)** Reducing the size of the ADC using nanobody ADCs may help to increase perfusion of the drug into the poorly vascularized and desmoplastic tumor microenvironment (TME) associated with pancreatic cancer. **(C)** Targeting TME associated antigens may bypass the poor internalization associated with tumor antigens but still allow tumor specific delivery of the payload. **(D)** Targeting two different mechanisms of cell death by combining ADCs with chemotherapy such as gemcitabine, may help to overcome resistance and improve efficacy compared to either treatment alone. ***ADC***: *antibody drug conjugate;****CAF***: *cancer associated fibroblast;****DC***: *dendritic cell;****GPC-1***: *glypican-1;****PD-1***: *programmed cell death protein 1*

Although preclinical studies have shown promise for the therapeutic application of ADCs for the treatment of pancreatic cancer, clinical trials so far have shown limited efficacy as a monotherapy. This is likely due to several factors which include the characteristics of the ADC itself such as choice of tumor antigen, linker molecule or payload. Another limitation of these pre-clinical studies, which makes assessment of clinical effectiveness difficult is that most studies have employed a human antigen-specific antibody, which targets the xenograft tumours, but does not bind to the equivalent antigen expressed by healthy tissues of the murine hosts, and thus does not represent the toxicities of off-tumor binding of the ADC. Moving forward, it is important that there is a focus on developing therapeutic strategies that address and overcome some of the barriers facing ADCs in pancreatic cancer, including target selection, ADC characteristics and therapeutic combinations (Fig. 2).

Targeting the right tumor antigen is a crucial factor to the success of an ADC. Given the vast array of targets being investigated in pancreatic cancer, it would suggest that we are yet to determine the best target for this disease. Some studies noted that antigen expression does not necessarily correlate with clinical response [88], and suggest that poor antigen internalization and trafficking of the ADC into tumor cells may be limiting factors to their effectiveness in patients [88, 90]. Approaches that target the tumor stroma rather than cancer cells, e.g. glypican-1 on CAFs [56] or tumor endothelial marker 8 (TEM8) on CAFs, endothelium and pericytes [92] present as a promising approach moving forward. These strategies may help to bypass the poor internalization and trafficking of tumor associated antigens and deliver free drug to the tumor microenvironment in a tumor specific, target independent manner.

The pancreatic tumor microenvironment, which is known to be extremely desmoplastic, poorly vascularized and immunologically suppressed, may also contribute to the limited efficacy of ADCs in the clinic. As an ADC is composed of an intact antibody, the ADC may poorly perfuse into the tumor due to its large size, limiting access to tumor associated antigen. New research approaches are focussing on reducing the size of these molecules to improve their pharmacological properties [93]. A recent study developed a single domain or nanobody ADC targeting the oncofetal antigen 5T4, conjugated to the topoisomerase inhibitor SN-38 [94]. The authors demonstrated using both tumor organoids from pancreatic cancer patients and pancreatic cancer xenograft models, that the nanobody ADC demonstrated superior tumor penetration, higher tumor uptake and faster accumulation compared to a conventional IgG1-based ADC.

Combining drugs that work by different and complementary mechanisms of action, has proven to be an effective strategy for targeting cancer. Resistance to gemcitabine chemotherapy is common in pancreatic cancer and as a result combining gemcitabine with other chemotherapeutics such as nab-paclitaxel have shown to improve therapeutic benefit [5]. The combination of gemcitabine with ADCs has been investigated in a number of preclinical studies [92, 95, 96] and the combination is now being tested in clinical trials (NCT04659603, NCT03816358). Cazes et al. investigated TR1801-ADC, an ADC which targets the tumor antigen MET and is conjugated to the PBD toxin, tesirine, in human pancreatic cancer xenograft models. They found that TR1801-ADC was able to synergize with gemcitabine and improve tumor response in gemcitabine-resistant PDX tumors [95]. Similarly, MLN0264 an anti-GCC targeting ADC showed superior tumor growth inhibition when combined with gemcitabine, compared to either therapy alone in pancreatic PDX models [96]. Szot et al. also demonstrated significantly reduced tumor burden in orthotopic human pancreatic cancer models when their TEM8 + stoma targeting ADC was combined with gemcitabine compared to either treatment given as a monotherapy [92]. The combination of gemcitabine with ADCs is now being tested clinically with tusamitamab ravtansine, an ADC selectively targeting CEACAM5, currently being evaluated alone and in combination with gemcitabine in patients with metastatic pancreatic adenocarcinoma (NCT04659603). The mesothelin-targeting ADC, AR, is also being evaluated in combination with gemcitabine and nivolumab (NCT03816358). Early data is showing promising results for the AR combination, with all eight patients treated so far achieving stable disease [83].

Pancreatic cancer is commonly associated with a highly immunosuppressive microenvironment and consequently limited efficacy with immunotherapy has been seen. Pancreatic cancer is associated with low mutational burden, leading to reduced antigenicity and allows the cancer to escape immunosurveillance [97]. The low antigenicity of pancreatic cancer can also be attributed to reduced DC numbers and perturbed antigen presentation [98]. Defects in the maturation of DCs in pancreatic cancer patients have also been described and is suggested to contribute to tumor tolerance [99]. The combined cytotoxicity and immunomodulatory properties of ADC payloads may help to overcome poor antigenicity in pancreatic cancer not only by inducing DNA damage but also by increasing DC maturation and upregulation of MHC antigen presentation machinery, which has been described for a number of ADCs [19–21, 100]. The immune-suppressive pancreatic tumor microenvironment is also attributed to the extensive infiltration of M2-type macrophages, myeloid derived suppressor cells (MDSC) and T-regulatory cells coupled with reduced numbers of cytotoxic T lymphocytes [101, 102]. Pro-tumorigenic M2 macrophages contribute to the immune-suppressive microenvironment through the secretion of anti-inflammatory cytokines and proteases and by promoting the expansion of MDSC, which potentiate the immune suppression by recruiting T regulatory cells to inhibit CD8 + effector function [103]. Interestingly, unlike DCs the immunomodulation of macrophages by ADCs is poorly understood. There is evidence that chemotherapeutic agents with similar mechanisms of action to ADC payloads such as cyclophosphamide and paclitaxel can influence macrophage phenotype and activation by promoting an M1 pro-inflammatory phenotype [104, 105]. ADC mediated conversion of macrophages toward a pro-inflammatory and anti-tumorigenic state, would contribute to overcoming the immunosuppressive TME in pancreatic cancer and further investigation into the immunomodulatory properties of ADC in this area is warranted. Combining ADCs with immune checkpoint inhibition represents a rational therapeutic strategy, owing to these immunomodulatory properties of ADC payloads and the potential for synergy with immunotherapy. Hence, combination studies have become a central focus of ADC studies both preclinically and clinically. Although ADCs and checkpoint inhibitors have been trialled in several cancers including breast, urothelial, lung and ovarian cancer [106], there are limited studies in pancreatic cancer. Pancreatic cancer is known to be highly immune-suppressed with limited activity seen with immunotherapy alone [107]. MGC018 is a duocarmycin-based ADC that targets B7-H3, an immunomodulatory molecule that is overexpressed on a wide range of cancers including pancreatic cancer. In preclinical studies, MGC018 was shown to be effective against pancreatic PDX models and in immunocompetent mouse models expressing human B7-H3, with the combination of MGC018 with anti-PD-1 checkpoint inhibition being synergistic [108]. MGC018 is currently being evaluated in a phase I clinical trial (NCT05293496) in combination with a bispecific DART molecule that targets both CTLA-4 and PD-1 (lorigerlimab), in solid tumors including pancreatic cancer. Other ADCs are also being evaluated clinically in pancreatic cancer patients in combination with checkpoint inhibition including XB002 (NCT04925284) and AR (NCT03816358). These trials are ongoing, so it remains to be determined if this combination will be successful for pancreatic cancer.

Surgical resection remains the only curative option for pancreatic cancer patients, however less than 20% of patients are eligible at diagnosis. Neoadjuvant therapy is used in cases of advanced but locally confined disease to control the tumor prior to surgical resection. In pancreatic cancer, neoadjuvant therapy is recommended by International Guidelines for borderline resectable and locally advanced pancreatic cancer [109]. The aim of neoadjuvant therapy is to prolong survival and to increase resection rates in patients with more advanced disease by controlling early systemic spread and increasing tumor-free resection margins [110]. Neoadjuvant therapy in pancreatic cancer has focussed on chemotherapy or chemoradiotherapy, but in other cancer types, ADCs are being explored in this space. The HER2 targeting ADC trastuzumab emtansine in combination with pertuzumab has been shown to be as effective as neoadjuvant chemotherapy in HER2 + breast cancer and was associated with a reduced toxicity profile [111–113]. Trastuzumab deruxtecan is also being evaluated as a neoadjuvant therapy low breast cancer with low HER2 expression (NCT04553770) [114]. Trop-2 targeting ADC sacituzumab govitecan has shown success as a single agent neoadjuvant therapy in triple negative breast cancer patients (NCT04230109) [115]. Brentuximab vedotin has been used successfully as a neoadjuvant in lymphoma [116] and enfortumab vedotin has shown efficacy in invasive urothelial cancer [117]. Given the promise in other cancer types, there is certainly potential for the use of ADCs as neoadjuvant therapy in pancreatic cancer.

## Conclusions

Pancreatic cancer is one of the most lethal malignancies, with very limited treatment options for the majority of patients. Through tumor specific delivery of highly potent cytotoxic agents, ADCs present as an attractive therapeutic approach for the treatment of pancreatic cancer. This is demonstrated by the large body of pre-clinical studies showing proof of anti-tumor activity through the targeting of a range of different tumor associated antigens in pancreatic cancer. Despite a number of clinical trials being undertaken to evaluate ADCs in pancreatic cancer, the clinical translation of this pre-clinical promise is yet to be realised. Factors including the harsh TME, altered vascularization of pancreatic tumors as well as poor antigen mediated ADC internalization are some of the main hurdles faced by ADCs in pancreatic cancer and further investigation to evaluate the molecular mechanisms of intrinsic and/or acquired resistance to ADCs in pancreatic cancer is warranted. Nonetheless, this versatile and diversifying therapeutic platform provides endless opportunities for customization and fine-tuning for improved tumor targeting, linker chemistries and payload properties. Future work in this area focused on tailoring the ADC to better tackle the pancreatic cancer microenvironment, will likely increase ADC effectiveness. Similarly, rationally designed combination therapies also hold great potential to improve efficacy and potentiate anti-tumor immune response in pancreatic patients and we await results from ongoing trials to guide future research in this area. While ADCs have not yet hit the mark in pancreatic cancer, the constantly evolving nature of this treatment modality means that there is still potential for ADCs to have a relevant clinical impact in the treatment of pancreatic cancer.

## Data Availability

Not applicable.
